# Selecting the Best Surgical Treatment Methods in Oro-Antral Communications

**DOI:** 10.3390/ijerph192114543

**Published:** 2022-11-05

**Authors:** Despina Luciana Bereczki-Temistocle, Simona Gurzu, Ioan Jung, Adina Cosarca, Gabriela Beresescu, Vlad Golu, Cecilia Petrovan, Alina Ormenisan

**Affiliations:** 1 Department of Oral and Maxillo-Facial, George Emil Palade University of Medicine, Pharmacy, Science, and Technology of Targu Mures, 540142 Targu Mures, Romania; 2 Department of Morphopatology, George Emil Palade University of Medicine, Pharmacy, Science, and Technology of Targu Mures, 540142 Targu Mures, Romania; 3 Department of Tooth and Arch Morphology, George Emil Palade University of Medicine, Pharmacy, Science and Technology of Targu Mures, 540142 Targu Mures, Romania

**Keywords:** Bichat fat pad flap, communication, buccal flap, general conditions, relapse

## Abstract

Introduction: an oro-antral communication is defined as a permanent pathological connection between the maxillary sinus and the septic oral cavity. Several flaps can be used for the closure (buccal flap, palatal flap, combination techniques) but relapses occur often in case of a large defects and underlying general conditions. Bichat fad pad flap is a multipotent pedicled fatty tissue that is easily accessible from the oral cavity that can be used for the closure of medium-sized defects, even in immunocompromised patients due to its stem cell capacity. Materials and methods: the medical information of the patients diagnosed with oro-antral communications who were admitted and treated in the Oral and Maxillo-Facial Clinic Targu Mures, between 2013 and 2020 were analyzed. A database containing general information, reported causes, associated diseases, surgical methods used during admission, and relapses, was created. The information was statistically processed. The written consent and ethical approval were obtained. Results: the study shows that from a total of 140 cases, 72 were treated using buccal advancement flap, 49 using Bichat fat pad flap, and 19 using palatal flaps. The dimensions of the communications ranged between 0.3 cm and 1.5 cm. Several statistically significant results could be found when comparing the surgical methods. Of the 72 patients treated with buccal advancement flaps, 25 presented relapses as opposed to the patients treated with Bichat fat pad flaps who showed no complications, *p* < 0.05. Analysing this aspect further, all large defects (10 cases) ranging from 0.6 cm to 1.5 cm treated with advancement buccal flaps (Rehrmann flaps) showed relapses (*p* < 0.05). Considering the general conditions, out of 7 patients who received radiotherapy 4 presented relapses, as opposed to the healthy patients, *p* < 0.05. Regarding the reintervention for the relapsed cases, the majority of the cases treated a second time with buccal advancement flap (5 out of 7 cases) failed as opposed to the Bichat fat pad flap with no further relapses (*p* < 0.05). Conclusions: the most frequently used surgical treatment is the buccal flap, which also has the highest relapse rate. Both primary treatment with Bichat fat-pad flap and re-treatment of relapses using this flap have had 100% success rates, even in patients with general associated conditions, in contrast with patients treated by using the buccal flap. The dimensions of the oro-antral communication and general conditions are crucial factors for the success of the surgical treatment.

## 1. Introduction

The oro-antral communication is defined as an unnatural communication between the maxillary sinus and the oral cavity. It is one of the most frequently occurring accidents during the extraction of maxillary molars and premolars (teeth with proximity or even projection of the roots within the maxillary sinus) [1].

In the era of fixed dentures on implants, oro-antral fistulas may develop after sinus lift surgery and improper implant placement [2].

In addition to the iatrogenic causes, oro-antral communication can also occur after trauma, infectious complications, or even tumours that in evolution destroy the maxillary bone and cause permanent connections between the sinus and the oral cavity [3].

Medication induced osteonecrosis is gaining more attention in the medical community, because of the complex evolution, affecting not only the local vascular supply but also the bone cell lines. Studies try to find the best medical and surgical treatment course to improve patients’ outcomes. In this context, the buccal fat pad flap has been successfully used for osteonecrosis induced oro-antral communication [4].

The smaller defects <2 mm can heal spontaneously by blood clot development and secondary healing, whilst larger defects require surgical treatment. Defects over 3 mm should be surgically treated to avoid further complications [1].

Undiagnosed and untreated in time, because of the constant contact with the oral cavity and because of improper sinus drainage, infections are quite common. Local manifestations include swelling, mucosal erythema, large amounts of granulation tissue inside the tooth alveoli and surrounding the antral fistula, purulent fluid accumulation in the sinus cavity. General examination usually reveals fever, fatigue, loss of appetite. These signs and symptoms describe an associated acute sinusitis. In these situations, antibiotics must be prescribed to treat the infections along with anti-inflammatories, antialgics and possibly endoscopic drainage of the sinus. Even if the infection is eradicated, treatment of the cause is mandatory. If the oro-antral communication is not surgically sealed, a permanent oro-antral fistula will develop and the sinus mucosa will transform into non-self-developing polyps, ulcerations, epithelial metaplasia, fibrosis. These changes are specific for chronic oro-antral fistulas and chronic sinusitis. In chronic cases, the general state of the patient is slightly affected but a multitude of signs and symptoms arise, such as nasal fluid regurgitation, pain, nasal obstruction or discharge, and cacosmia. Implementing a correct and rapid treatment, adapted to each particular clinical situation is essential in order to prevent infection and dissemination. Along with the surgical treatment of the oro-antral communication sinus irrigation or even radical sinus surgery, Caldwel-Luc intervention must be performed [1,2,3,4].

The purpose of this article is to compare different surgical treatment methods currently used to close oro-antral communications, to determine relapse rates in case of different types of flaps used, the advantages and disadvantages of each therapeutic solution, in order to assess the best treatment option. The size of the defect, the anatomical particularities, the existing general conditions, and further complications of the oro-antral communication, must be considered to obtain a complex treatment plan. Moreover, particularly important in developing the correct treatment plan is radiological examination. Computed tomography (CT) or cone beam computed tomography (CBCT) evaluates not only alveolar and the sinus floor defect, but it also presents the mucosal transformations [5,6].

There is a variation of loco-regional flaps that can be used in closing oro-antral communications, depending on the dimension of the defect and the available soft tissue surrounding the dental alveoli. For a proper wound closure, local flaps should consist of healthy tissue, without pathological modifications and should present a sufficient blood supply to promote healing. There are two most used flaps, those being the buccal advancement flap and the palatal rotational flap [5]. Recent studies recommend the use of the Bichat buccal fat pad flap for the closure of oro-antral communications [5,6].

Currently, the literature describes using collagen membranes and bone grafts for the closure of oro-antral communications with satisfactory results [7].

The buccal advancement flap, the most commonly used flap by dentists and dental surgeons for the closure of oro-antral communications, is created by placing a horizontal incision in the gingival sulcus and two divergent buccal vertical incisions, obtaining a trapezoidal flap. In most of the cases periosteal scoring is necessary to mobilize the flap [8]. It is a simple and quick technique, which does not need special manoeuvres to create it. The disadvantages include a reduced vestibule and, in case of larger defects, wound dehiscence because of the affected blood supply in case of supplemental scoring. In addition, a constant traction is exercised on the flap during mastication and phonation, which may affect the healing process [9].

Palatal advanced and rotated flap is another surgical option. A full thickness flap that contains the palatal artery is a good option to close an oro-antral communication, but the main disadvantage is the limited mobility of the flap. It forces the surgeon to create a large palatal defect with further extensive scarring. Donor site morbidity is frequent, and the discomfort of patients is considerable [5].

There are several uses for the adaptable fatty tissue known as the Bichat fat pad flap. Initially, the buccal fat-pad flap was utilized to repair medium-sized defects following oral cancer surgery. The fat cells act as a stem cell, and this flap may change into any kind of tissue given the right circumstances. When used to close oral wounds, it transforms into healthy oral mucosa (squamous epithelium). It consists of a main body of fat, with the buccal, pterygoid, superficial, and deep temporal extensions. The average volume is 9.6 mL and it is known to remain constant regardless of age and gender. It has a rich blood supply, being irrigated by the three arteries of the subcapsular plexus: maxillary, superficial temporal, and facial artery. The role of the buccal fat pad is to ensure the protection of the neurovascular bundles and ensure intermuscular movement. The most superficial part is the buccal one, which can be accessed below the created flap, by placing a small incision in the periosteum, to close oral defects located in the posterior part of the maxilla. The Bichat fat-pad is exposed and advanced towards the defect gently and sutured to the palatal mucosa without tension [10].

## 2. Materials and Methods

This retrospective study was conducted between January 2013 and December 2020 at the Oro-Maxillo-Facial Surgery Clinic in Targu Mures, Romania which is a tertiary centre and academic hospital.

A total of 140 patients diagnosed with oro-antral communication associated or non-associated with chronic sinusitis were included in this study. Other analysed data were the cause of the defect, dimensions of the communication, type of surgical closure, relapses, reintervention, and eventual second relapses.

The study was conducted according to the guidelines of the Declaration of Helsinki and approved by the Ethics Committee of the George Emil Palade University of Medicine, Pharmacy, Sciences, and Technologies of Targu Mures, Romania (approval number 32647/13.12.2018). Informed consent was obtained from all subjects involved in the study.

We reviewed the charts and release papers of every patient with the afore-mentioned main diagnostic and collect general information regarding the patients: name, gender, age, and associated general conditions.

Following the surgical operation, controls were carried out for removal of sutures and then every six months, during a period of at least 2 years. We initially identified 289 patients who had been treated with the aforementioned flaps, but 149 did not return for frequent check-ups and, thus, were eliminated from the research.

All the information was processed and analysed, creating a database using Microsoft Excel. Statistical analysis was performed using GraphPad Instat 3 trial and SPSS.

## 3. Results

Out of the total of 140 patients included in the study, most of them were male (88) and 52 were female. Regarding the age of the patients diagnosed with oro-antral communications, there were three age categories: young patients (age 20–30), adult patients (age 31–60), and elderly (over 61 years old).

Table 1 shows the main causes of oro-antral communication and range of dimensions.

Of the 140 patients treated using buccal advancement flap, Bichat fat pad flap, and palatal flap, almost half presented associated chronic sinusitis (Table 2).

**Figure 1:**
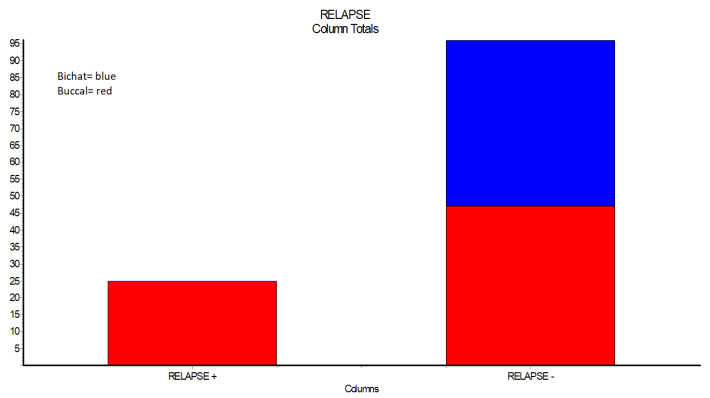
Relapse in case of surgical closure with Bichat fat pad flap and case of closure with Buccal advancement flap.

None of the 49 cases treated with Buccal fat pad flap showed any relapses or complications. From the 72 cases treated with buccal advancement flaps, 25 presented dehiscence, *p* < 0.05 (Table 3, Figure 1).

**Figure 2:**
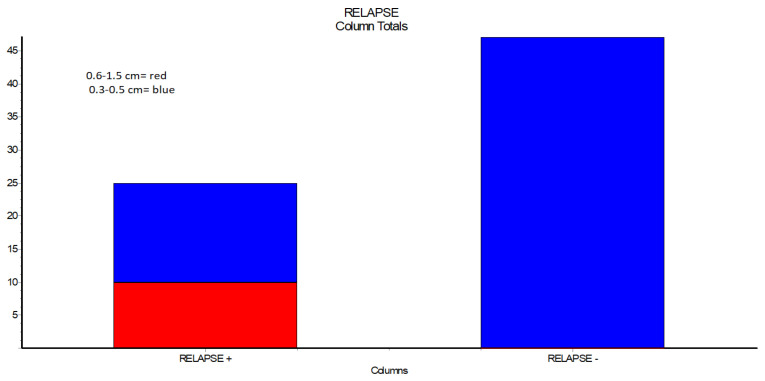
Relapses in case of closure with buccal advancement flap, the outcome for small defects and large defects.

Regarding relapses in case of buccal advancement flaps, larger defects (0.6–1.5 cm) were noted in 10 out of 10 cases presented by relapses (Table 4, Figure 2).

**Figure 3:**
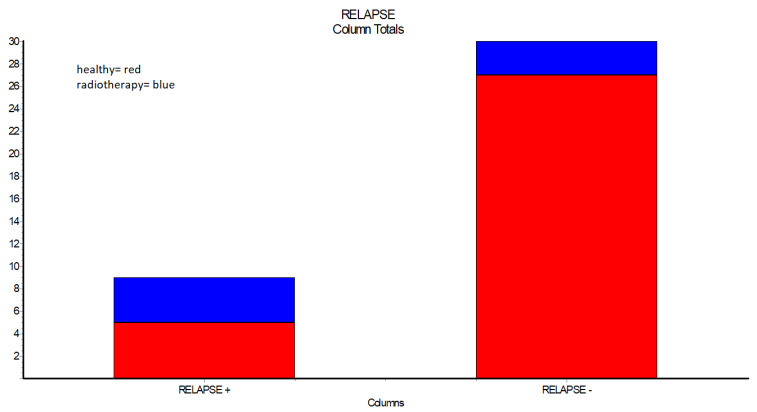
Occurrence of relapses in healthy patients and patients with a history of radiotherapy.

Further examination of the cases showed that out of 7 patients who received in past radiotherapy, more than half presented relapses when compared to healthy individuals (Table 5, Figure 3).

Smoking was considered as a risk factor for improper healing of the wound after reconstruction (Table 6).

For the cases that presented reoccurrence of the oro-antral communication, reintervention was performed using in most of the cases Bichat fat pad flap, and some using again buccal advancement flap. Out of 7 cases involving buccal flap, approx. 70% failed. All Bichat flaps were successful. Here, 3 patients did not benefit from the reintervention surgery in the OMF Clinic Targu Mures. From 29 initial relapses, 26 cases remained for further analysis (Table 7).

When comparing the flaps, there were better outcomes for the palatal flap than the buccal advancement flap, although, not statistically significant (Table 8).

## 4. Discussion

The findings of our investigation show that males account for two-thirds of the 140 patients with oro-antral communications.

We found that more than 60% of the patients with oro-antral communication that were between the ages of 30 and 60, with just 20% of them being young patients between the ages of 20 and 30. These findings are explained by the study of Park J et al., which identified a mean age of 54.8. Sinus architecture and development also contribute to this explanation [11].

Patients hospitalized to the Oral and Maxillofacial Clinic in Targu Mures were diagnosed with oro-antral communications following cystectomy, poor implant placement, and bone augmentation. However, most of the defects (97 out of 140 patients) were caused by the extraction of upper molars that were in direct contact with the sinus mucosa or had a thin bone plate separating the roots from the sinus mucosa. The literature supports the primary reason reported in this investigation [5].

Of the 140 patients, 74 had chronic sinusitis and 60 had little sinus involvement or no sinus infection at all. Chronic sinusitis is usually diagnosed when symptoms such as nasal obstruction, nasal discharge, and relative discomfort in the afflicted sinus remain for more than 12 weeks. Due to this, the current study may be able to estimate the time between the onset of oro-antral communication and the surgical closure of the lesion. That indicates that over half of the patients received treatment after a minimum of 12 weeks. Existing procedures describe that in chronic situations, the sinus mucosa becomes a non-self, with no way to correct chronic epithelial metaplasia, polyps, cystic lesions, or chorion fibrosis. Even if the oro-antral connection is closed, the underlying sinus infection must be treated with a combination of drastic surgical surgery to remove the altered mucosa and general antibiotic, anti-inflammatory, and antalgic treatment [4].

The surgical closure approach to oro-antral communication is the subject of the investigation. The Bichat fat pad flap produced the best results in 72 cases, despite the buccal advancement flap being the most popular flap—a finding that is supported by the literature [12]. The 49 instances that were successfully treated using the fat pad flap had no dehiscence, no local infection, or any problems. Regarding the buccal advancement flap, the same problem was retreated in 25 cases. Fisher’s exact test statistical analysis yielded a very significant result of *p* < 0.0001. The Bichat fat pad flap is a viable therapeutic option with excellent local healing, according to a study published in 2021. It can also be utilized effectively in a single layer without requiring additional surgery and without altering the sulcus’s depth. It is not necessary to apply the buccal flap in contact with the palatal ridge, thus permitting the practitioner to suture it in a higher position [13].

The benefits of the Bichat fat-pad flap over the use of a buccal flap have not been proven in other investigations, such the one carried out by a team of researchers in India in August 2018 [14]. Marci H. Levine and Silvia Spivakovsky made the same claim in their 2017 article, “Low quality evidence for treatment options for oro-antral communications” [15].

In terms of reintervention for relapsed cases, three patients at the Oro-Maxillofacial Clinic Targu Mures did not complete the second treatment. Further analysis was performed on the remaining 26 patients out of 29 who showed residual oro-antral communications after the first attempt. The Bichat fat pad flap was used in 19 of the 26 cases with dehiscence and the buccal advancement flap in 7 cases. There were no second relapses in the first group, but 5 in the second group were unsuccessful (*p* < 0.003). These findings support the notion that the fat pad flap is a dependable treatment option, even in the face of previous failure. The reintervention with the buccal advancement flap may have failed due to the scarred buccal flap’s poor quality. General conditions and dimension of the oro-antral communication may potentially provide unfavourable conditions for healing. Similar findings were found in a study of Mahamadou Konate et al., that sustain the fact that the buccal fat pad flap has promising results, but that it may present necrosis if used in a single layer. In our study all of the patients were treated with Bichat fat pad flap in a single layer, thus avoiding the reduction of the vestibule, without any signs of necrosis [9]. One article recommends using a double layer technique using septal cartilage for the bone defect and Bichat buccal fat pad with favourable results [16]. Other evaluations of the literature concluded that both treatment approaches are viable, with no relapses; however, present trials use small groups, and investigators recommend that larger groups be included in future trials for more reliable results [17]. The current study was carried out over a 7-year period, to assess the evolution, postoperative controls were carried out for removal of sutures and then every six months. When recall in our unit was not feasible (e.g., distant place of residence) controls were carried out by the local dentist. Readmissions for the same pathology were taken into consideration to identify potential relapses.

Following a closer review of the data, particularly the relapses in the case of buccal advancement flaps, a statistical comparison shows that the oro-antral communication’s dimension is a crucial element that needs to be considered. The buccal flap has a better success rate when defects are modest, ranging from 0.3 cm to 0.5 cm in this study, than when defects are larger, 0.6 cm to 1.5 cm, *p* < 0.0001. Since blood flow is restricted by scoring the periosteum, the extent of the oral defect must be carefully considered when utilizing a single-layer buccal advancement flap, which is a full-thickness trapezoidal flap. This conclusion is supported by a study by Shukla B et al. from 2021, which emphasizes the importance of the size and location of the defect in the treatment of oro-antral communication and claims that the Bichat fat pad flap is a superior treatment choice due to the complex blood supply and low donor site morbidity [18].

Another assessed treatment option was the palatal flap. In this case, 19 patients from this database were treated using palatal rotational flaps. Although when comparing the palatal flap to the buccal flap, there is a better outcome that can be seen for the first option, the results were not statistically significant. The lack of flexibility of the palatal flap, forces the surgeon to create a large incision to assure the rotation of sufficient tissue. Furthermore, the palatal flap has a great blood supply and maintains texture quality, but the disadvantages are increased donor site morbidity and postoperative discomfort of the patient. It can be used according to the literature for smaller defects corresponding the premolar region [19]. Studies conclude that the buccal advancement flap has a higher success percentage than the palatal flap [19,20].

Considering all three flaps, the general conclusion of several studies is that the Bichat fat pad flap has the highest success rate [17,19,20].

Patients who experienced relapses had a variety of general health issues and risky behaviours, including radiation, cigarette and alcohol use, diabetes, cardiovascular disease, kidney impairment, and other general health issues. Only one case with medical induced osteonecrosis and oro-antral communication was identified.

No significant differences were identified between healthy individuals and those consuming alcohol, or those with controlled general conditions, including Diabetes mellitus, but there were important implications in patients who had a history of radiotherapy. Patients diagnosed with head and neck cancer receive as part of the complex treatment of malignancies radio- and chemotherapy. The impact of this treatment is general and local, and determines weight loss, malnutrition, weakening of the immune system, hypo-salivation, dysphagia, mucosal atrophy, infection, periodontal disease, increased cavity susceptibility, and osteonecrosis. Radiotherapy disrupts the local healing process, affecting also healthy tissue and young cells. Chronic vascular disease is also a frequent complication of radiotherapy. The blood vessels present atherosclerosis, stenosis, and thrombosis. That is why any surgical intervention may result in complications, lack of wound healing or bone regeneration [21].

Numerous studies over the years have proven that radiomucositis is a grave consequence of radiotherapy [22]. Oral mucositis refers to erythematous and ulcerative oral lesions frequently encountered in cases of patients with a personal history of radiotherapy. These lesions are painful and affect the quality of life of the patient, and their nutritional status and contribute to local and systemic infection by lowering the capacity of their immune defence system [23].

This paper shows a statistically significant difference between healthy patients and patients with a history of radiotherapy regarding relapses. From 7 patients that received in the past radiotherapy, 4 present relapses after surgical closure of the oro-antral communication (*p* < 0.0370).

Nicotine slows down the healing process of both connective tissue and osteogenesis, interfering also with the angiogenesis in the ossification process and healing of the mucosa. Some studies show that there is a correlation between nicotine dosage and a prolonged healing period. Furthermore, nicotine inhibits the proliferation of fibroblast cells, and their adhesion and reduces collagen synthesis. Its vasoconstrictor effect can predispose to microvascular thrombotic obstruction, followed by ischemia [24].

Smoking is typically thought to be a risk factor for oral bacterial and fungal infection, as well as poor wound healing. The present study found no evidence that smoking was a favourable factor for the failure of oro-antral communication surgery, and there were no discernible differences between non-smokers and smokers. This is a result supported by Sella A. et al.’s research. The outcomes of this study revealed that there is no difference in failure rates between smokers and non-smokers, although smokers feel more pain and edema after surgical procedures [25].

The optimum treatment plan must consider a variety of aspects in context of these findings. The dimension, position of the communication, associated general conditions of the patients, associated sinus infection, postoperative instructions, and care are all critical for success [6].

Initially, the Bichat fat pad flap was used for small and medium-sized defects, after malignant and benign tumour excisions. The limited availability of the oral soft tissue challenged practitioners to investigate and to find a solution that provided full closure of the defect using a high-quality flap with proper blood supply. The Bichat fat pad flap has high success rates in cancer patients even if previous flaps fail. An important advantage in these cases is that radiation treatment is not considered a contraindication [26]. Furthermore, this innovative flap has several other applications discovered over the years. It can be successfully used in congenital defects, such as cleft palate, on raw bone surfaces assuring a proper healing and preventing palatal fistulas and avoiding velopharyngeal insufficiency. Other uses of the BFP include TMJ restoration, in which a limited gap of 6–7 mm is advised for the arthroplasty, enhancement of the upper lip profile following maxillary advancement surgery by Le Fort I osteotomy, repair of the skull-base defected following tumour excision, avoidance of Frey syndrome following parotid surgery, repair of the maxillary sinus membrane post-dental implant, and vocal cord augmentation [10].

The Bichat fat pad flap requires a small incision without damaging the surrounding tissues and an experienced surgeon would consider this flap as a versatile solution for the closure of medium size defects approx. 4 cm diameter wounds without major complications and with a great success rate [10,20].

Some studies provide a contemporary alternative to soft tissue closure for addressing oro-antral communication while preserving the possibility of placing dental implants and restoring the functionality of the damaged area. A study from 2021 proposed a sinus bone graft employing a collagen membrane in the shape of a pouch fixed by bone tracks and the defect filled with allografting material and closed with another membrane, a periosteum-releasing incision to allow the buccal flap to close predominantly. After 6 months, they discovered that appropriate bone dimensions had been obtained, with no sinus problems or relapses of oro-antral communication [7,27].

We believe that this flap is the greatest alternative for oro-antral communications, even after unsuccessful implant placement and earlier closure efforts, and even in immunocompromised individuals, with data indicating no relapses and no problems.

## 5. Conclusions

According to the present study, we conclude that:

o Most patients with oro-antral communications are male.
o Age and controlled general conditions do not influence the case evolution.
o Palatal flaps show slightly better results as buccal advancement flaps, but the results are not statistically significant.
o The most frequently used surgical treatment is the buccal flap, which also has the highest relapse rate (*p* < 0.0001).
o Radiotherapy affects the success of the surgical closure (*p* < 0.0370).
o The buccal flap should be used for small defects (*p* < 0.0001).
o The Bichat fat pad flap is the best option for reinterventions (*p* < 0.0003).
o Bichat fat pad flap is the best treatment option for oro-antral communications even in case of associated general conditions, large defects, or relapses.

Further research is needed, including a greater number of patients treated with various flaps, to develop a thorough treatment plan that ensures minimal risks and optimum patient comfort.

## Figures and Tables

**Table 1:** Main causes: during bone graft placement (augmentation), cystectomy, implant placement, postexodontia (post extraction); dimensions: from 0.3 cm to 1.5 cm.

	Dimensions									Total
	CM	0.3	0.4	0.5	0.6	0.7	1.0	1.2	1.5	
Cause	Augmentation	0	0	1	0	0	1	0	0	2
	Cystectomy	3	2	10	3	2	5	3	3	31
	Failed implant	0	0	0	3	1	1	3	1	9
	Osteonecrosis	0	0	0	0	1	0	0	0	1
	Postextraction	28	21	16	6	12	9	3	2	97
	Total	31	23	27	12	16	16	9	6	140

**Table 2:** Flaps used: Bfpf (Bichat fat pad flap), P (palatal flap), Baf (Buccal advancement flap); associated sinusitis (1), no associated sinusitis (0).

	CLOSURE			Total
	Bfpf	P	Baf	
Sinusitis (0)	6	5	63	74
Sinusitis (1)	43	14	9	66
Total	49	19	72	140

**Table 3:** Crosstabulation between identified relapses for Bichat fat pad flaps and Buccal advancement flaps.

	Relapse +	Relapse −	Total
Bichat flap	0 (0%)	49 (40%)	49 (40%)
Buccal flap	25 (21%)	47 (39%)	72 (60%)
Total	25 (21%)	96 (79%)	121 (100%)

The two-sided *p* value is <0.0001, considered extremely significant; RR = 0.000.

**Table 4:** Crosstabulation for use of buccal advancement flap in case of defects ranged between 0.3 cm and 0.5 cm and in case of defects ranged between 0.6 cm and 1.5 cm.

	Relapse +	Relapse −	Total
Buccal 0.3–0.5 cm	15 (21%)	47 (65%)	62 (86%)
Buccal 0.6–1.5 cm	10 (14%)	0 (0%)	10 (14%)
Total	25 (35%)	47 (65%)	72 (100%)

The two-sided *p* value is <0.0001, considered extremely significant; RR = 0.2419.

**Table 5:** Crosstabulation for the impact of radiotherapy on relapse occurrence.

	Relapse +	Relapse −	Total
History of radiotherapy	4 (10%)	3 (8%)	7 (18%)
Healthy patients	5 (13%)	27 (69%)	32 (82%)
Total	9 (23%)	30 (77%)	39 (100%)

The two-sided *p* value is 0.0370, considered significant; RR = 3.657.

**Table 6:** Relapses in healthy patients and in patients who smoke.

	Relapse +	Relapse −	Total
Smokers	10 (14%)	31 (42%)	41 (56%)
Non-smokers	5 (7%)	27 (37%)	32 (44%)
Total	15 (21%)	58 (70%)	73 (100%)

The two-sided *p* value is 0.3979, considered not significant; RR = 1.561.

**Table 7:** Reintervention using Bichat fat pad flap and Buccal flap.

	Relapse2 +	Relapse2 −	Total
Reintervention Bichat	0 (0%)	19 (73%)	19 (73%)
Reintervention Buccal	5 (19%)	2 (8%)	7 (27%)
Total	5 (19%)	21 (81%)	26 (100%)

The two-sided *p* value is 0.0003, considered extremely significant; RR = 0.000.

**Table 8:** Crosstabulation for palatal flaps and buccal advancement flaps regarding relapses.

	Relapse +	Relapse −	Total
Buccal flap	25 (27%)	47 (52%)	72 (79%)
Palatal flap	4 (4%)	15 (16%)	19 (21%)
Total	29 (32%)	62 (68%)	91 (100%)

The two-sided *p* value is 0.4065, considered not significant; RR = 1.649.

## Data Availability

Data supporting reported results can be found by contacting Gabriela Beresescu, felicia.beresescu@umfst.ro.
